# Equivalent reduction of Escherichia coli by rinsing hands with cold and warm water

**DOI:** 10.3205/dgkh000527

**Published:** 2024-12-16

**Authors:** Romana Kordasiewicz-Stingler, Michael Reiter, Günter Kampf, Jürgen Gebel, Carola Ilschner, Miranda Suchomel

**Affiliations:** 1 Institute for Hygiene and Applied Immunology, Medical University of Vienna, Austria; 2 University Medicine Greifswald, Germany; 3 Institute for Hygiene and Public Health, University Hospital Bonn, Germany

**Keywords:** hand rinsing, water temperature, EN 1499

## Abstract

**Objective:**

Hand washing is considered an important public health intervention to reduce the burden of communicable diseases such as gastrointestinal and respiratory tract infections. Washbasins in public restrooms are often only equipped with cold water and it can be observed that people only rinse their hands briefly after using the toilet instead of washing them properly with soap. As there are no recommendations on the optimal water temperature for efficacy, we measured the efficacy of simple hand rinsing with cold (4°C) and warm (40°C) water for 10 and 20 seconds compared to the European Norm EN 1499 reference hand wash.

**Methods:**

A Latin square design was used with five treatment groups and three participants per group. Hands were contaminated by immersion in an *Escherichia coli* suspension. Before and after the respective treatment fingertips were sampled to obtain pre- and post-values. Pre- and post-values were averaged separately for each volunteer and the arithmetic means of all individual lg reductions were calculated and compared using Wilcoxon’s matched-pairs signed rank tests (one-sided, P<0.05). Post hoc test of differences between treatmets was done by Tukey’s honest significant difference tests, P<0.05 was considered significant.

**Results:**

Rinsing hands for 10 seconds with cold (1.93 lg) or warm water (2.01 lg), and for 20 seconds with cold (2.23 lg) or warm water (2.39 lg) was significantly inferior to the 1 minute reference hand wash with sapo kalinus (2.68 lg), but there were no significant differences between the use of cold or warm water in the pairwise comparison for both times. However, the duration seems to have an effect on the bacterial reduction as the differences between the hand rinsing times were significant for both temperatures.

**Conclusion:**

Rinsing hands with cold water was as effective as warm water. Its implementation in the community could save energy and resources without losing any efficacy.

## Zusammenfassung

**Zielsetzung:**

Händewaschen gilt als wichtige Maßnahme der öffentlichen Gesundheit, um übertragbare Krankheiten wie Magen-Darm- und Atemweginfektionen zu verhindern. Die Waschbecken in öffentlichen Toiletten sind oft nur mit kaltem Wasser ausgestattet und man kann immer wieder beobachten, dass sich Nutzer nach dem Toilettengang nur kurz die Hände abspülen, anstatt sie richtig mit Seife zu waschen. Da es keine Empfehlungen für die optimale Wassertemperatur für die Wirksamkeit gibt, haben wir vergleichend die Wirksamkeit des einfachen Händespülens mit kaltem (4°C) und warmem (40°C) Wasser für 10 und 20 s nach der Europäischen Norm EN 1499 gemessen.

**Methode:**

Fünf Behandlungsgruppen mit jeweils drei Teilnehmern pro Gruppe wurden im Lateinischen Quadrat untersucht. Die Hände wurden durch Eintauchen in eine *E. coli*-Suspension kontaminiert. Vor und nach der jeweiligen Behandlung wurden die Fingerspitzen beprobt, um Vor- und Nachwerte zu erhalten. Die Vor- und Nachwerte wurden für jeden Probanden getrennt gemittelt. Von allen individuellen lg-Reduktionen wurden die arithmetischen Mittelwerte berechnet und mit dem Wilcoxon-Vorzeichen-Rang-Test für den paarigen Vergleich verbundener Stichproben (einseitig, P<0,05) ausgewertet. Post-hoc-Tests der Unterschiede zwischen den Behandlungen wurden mit dem Tukey‘s Test durchgeführt, wobei P<0,05 als signifikant angesehen wurde.

**Ergebnisse:**

Das Händespülen für 10 s mit kaltem (1,93 lg) oder warmem Wasser (2,01 lg) und für 20 s mit kaltem (2,23 lg) oder warmem Wasser (2,39 lg) war signifikant schlechter als das 1-minütige Referenzverfahren der Händewaschung mit Sapo kalinus (2,68 lg), aber es gab keine signifikanten Unterschiede zwischen der Verwendung von kaltem oder warmem Wasser im paarweisen Vergleich für beide Zeiten. Die Dauer scheint sich jedoch auf die Keimreduktion auszuwirken, da die Unterschiede zwischen den Handspülzeiten für beide Temperaturen signifikant waren.

**Fazit:**

Das Händespülen mit kaltem Wasser war genauso wirksam wie mit warmem Wasser. Die Umsetzung im öffentlichen Raum könnte Energie und Ressourcen einsparen, ohne an Wirksamkeit zu verlieren.

## Introduction

Hand washing is considered an important public health intervention to reduce the burden of communicable diseases such as gastrointestinal and respiratory tract infections. A Cochrane meta-analysis revealed that an increase of compliance with hand washing reduced diarrhoea episodes in both child day-care centres in high-income countries and among communities living in low and middle income countries by about 30% [1]. A similar finding was reported for respiratory tract infections. A meta-analysis of randomized controlled trials described that a single hand hygiene event is associated with a 3% decrease in the daily probability of an acute respiratory infection [2]. The optimum duration of hand washing and water temperature are, however, under dispute. For public settings, 22 of 51 guidelines recommend a hand washing duration for at least 20 seconds [3]. But the water temperature is not part of recommendations, except that the WHO recommends not to use hot water [4]. In addition, the washbasins in public restrooms are often only equipped with cold water. Furthermore, it can be observed in these settings that people only rinse their hands briefly after using the toilet instead of washing them properly with soap [5]. There are no data or recommendations on the optimal water temperature for hand rinsing efficacy in community settings. The aim of the study was therefore to determine the efficacy of simple hand rinsing with cold (4°C) and warm (40°C) water for 10 and 20 seconds.

## Materials and methods

### Study design

The experiments were performed according to the European Norm EN 1499, which is an in-vivo laboratory model for measuring hand wash efficacy on artificially contaminated hands of volunteers [6]. The efficacy of rinsing hands with plain cold (4°C) and warm (40°C) water for 10 and 20 seconds was compared to the EN 1499 reference hand wash with sapo kalinus (20%) for 1 minute. A Latin square design was used with five treatment groups and three participants per group, each performing one of the five hand treatments in parallel. At the end of the fifth test run, each volunteer had used each treatment once. The study protocol was approved by the institutional ethics committee of the Medical University of Vienna (2051/2022), all 15 volunteers gave their informed consent. Exclusion criteria were: less than 18 years of age, pregnancy, skin breaks such as cuts, abrasions or other skin disorders on the hands. Nails were short and clean and the volunteers agreed not to take or use any antibacterial or antibacterial soap during the trials, starting one week prior to testing.

### Hand wash procedures

Hands were washed for 1 minute with non-medicated soft soap (sapo kalinus) and dried with paper towels, immersed in the *Escherichia (E.) coli K*12 (NCTC 10538) contamination fluid (3.7x10^8^ cfu per ml) up to the mid-metacarpals for 5 seconds with fingers spread, and then allowed to air dry for 3 minutes. Fingertips were then rubbed for 1 minute at the bottom of a petri dish (including the thumbs) containing tryptic soy broth (pre-values), one for each hand. For the EN 1499 reference procedure, hands were washed with 5 ml of sapo kalinus (20%) for 1 minute, followed by rinsing for 10 seconds under running tap water with the finger tips pointing upwards. The other four hand rinsing procedures were performed with water at exact temperatures of 4°C and 40°C, which was rinsed over both hands while the hands were rubbed during the application time of 10 and 20 seconds. Immediately after the respective application time, the fingertips of both hands were sampled as described above (post-values). All sampling fluids were diluted and cultivated on the surface of tryptic soy agar with sodium-desoxycholate which prevents the growth of resident microbial skin flora and incubated at 36±1°C for 48 hours. 

### Statistics

For statistical evaluation, all colony counts per mL sampling fluid were expressed as decadic logarithms. Pre- and post-values were averaged separately for each person. The arithmetic means of all individual lg reductions were calculated and compared using Wilcoxon’s matched-pairs signed rank tests (one-sided, P<0.05). Post hoc test of differences between treatments was done by Tukey’s honest significant difference tests, P<0.05 was considered significant.

## Results

Rinsing hands with plain water for 10 seconds with cold (4°C) or warm water (40°C) reduced *E. coli* by 1.93 lg and 2.01 lg, respectively. When hands were rinsed for 20 seconds with cold or warm water, the mean lg reductions were 2.23 and 2.39, respectively. Although the mean lg reductions at 10 and 20 seconds were significantly lower compared to the 1 minute reference hand wash with sapo kalinus (2.68 lg; P<0.05), there were no significant differences between the use of cold (P=0.927) or warm water (P=0.566) in the pairwise comparison for both handrinsing times. There were significant differences between hand rinsing for 10 or 20 seconds for each of the two water temperatures in the pairwise comparison (Table 1 (Tab. 1)).

## Discussion

Our results are in line with others who also did not find a significant difference of hand washing efficacy at different water temperatures [7], [8], although our data are the first ones obtained according to the European Norm EN 1499 in a Latin square design. Importantly, the volunteers used only plain water without soap with a continuous water flow suggesting that the use of liquid soap may not be necessary to achieve a 2.0 lg reduction in 10 seconds. Previous studies described lower effect in 10 seconds when liquid soap was used such as 0.5 lg with *E. coli* [9], between 0.7 and 1.2 lg with rotavirus [9] and 1.9 lg with *Serratia marcescens* [10].

The preferred use of cold water has been advocated already by Carrico et al. [11]. However, the duration of hand rinsing seems to have an effect on the bacterial reduction as the differences between the hand rinsing times (10 and 20 seconds) were significant for both water temperatures. In reality, however, the hand rinsing duration may be shorter than 10 seconds. An observational study showed that the mean hand washing duration after visiting a rest room was between 8.0 seconds (male subjects) and 8.8 seconds (female subjects) [6].

Hot water is not recommended for hand washing due to the potential for skin damage [12], [13]. Warm water of 44°C has been described to be more harmful to human skin than cold water of 4°C, because it significantly increases the transepidermal water loss and reduces the stratum corneum hydration, resulting in an impaired skin barrier function and increased skin dryness [14]. Higher water temperatures, e.g. during showering, were also associated with a higher degree of dermal absorption of disinfection by-products such as haloacetonitriles and chloral hydrate [15].

Higher water temperatures are also associated with higher energy consumption [7]. Rinsing hands with cold water, which we found to be as effective as warm water, could therefore potentially save energy and resources without losing any efficacy. The influence of water temperature after previous soap washing was not additionally investigated in this study and could be considered a limitation.

## Conclusions

Our contribution demonstrates that rinsing hands with cold water is as effective as warm water in reducing bacteria from contaminated hands. The use of cold water for community hand rinsing facilities may be an option for saving energy without compromising the microbiological efficiency.

## Notes

### Competing interests

The authors declare that they have no competing interests.

### Ethical approval

The study protocol was approved by the institutional ethics committee of the Medical University of Vienna (2051/2022).

### Funding

This study was supported by the research fund of the Institute for Hygiene and Applied Immunology, Medical University of Vienna, Austria.

### Acknowledgement

The authors would like to thank Michael Kundi from Department of Public Health, Medical University of Vienna.

### Data availability

The datasets used and/or analysed during the current study are available from the corresponding author on reasonable request.

### Authors’ ORCID

- Michael Reiter: 0000-0002-7435-7999
- Günter Kampf: 0000-0003-2621-6419
- Jürgen Gebel: 0000-0001-9328-3174
- Miranda Suchomel: 0000-0001-8758-9652

## Figures and Tables

**Table 1:**
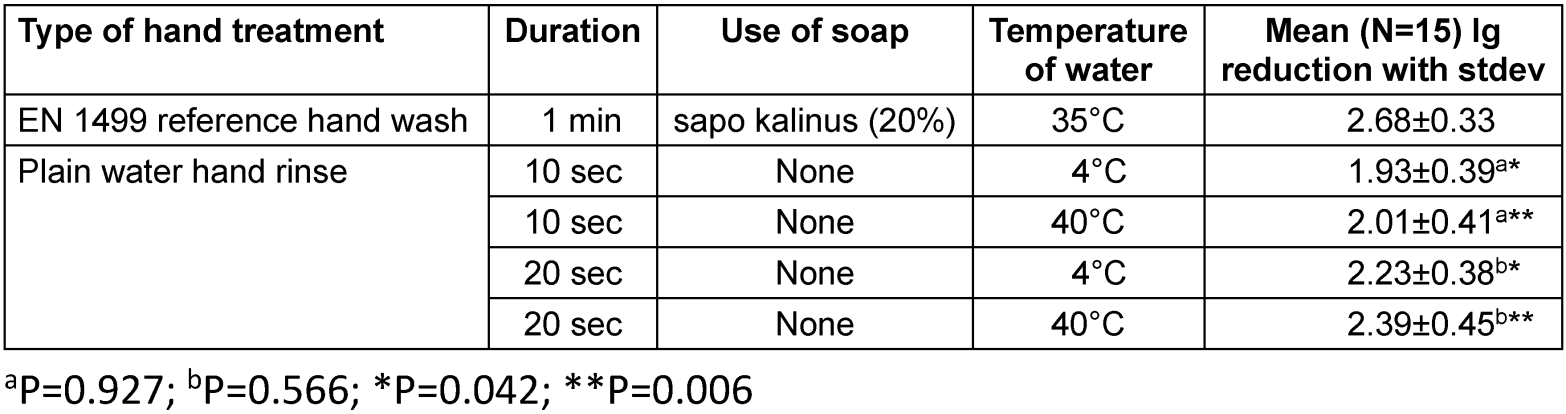
Mean lg reduction of *E. coli* on artificially contaminated hands by the EN 1499 reference hand wash and four different hand rinse procedures
